# Frost prediction using machine learning and deep neural network models

**DOI:** 10.3389/frai.2022.963781

**Published:** 2023-01-12

**Authors:** Carl J. Talsma, Kurt C. Solander, Maruti K. Mudunuru, Brandon Crawford, Michelle R. Powell

**Affiliations:** ^1^Los Alamos National Laboratory, Earth and Environmental Sciences Division, Los Alamos, NM, United States; ^2^Carbon Solutions LLC, Bloomington, IN, United States; ^3^Pacific Northwest National Laboratory, Watershed and Ecosystem Science, Richland, WA, United States; ^4^Los Alamos National Laboratory, Facility System Engineering Utilities and Infrastructure Division, Los Alamos, NM, United States

**Keywords:** frost damage, machine learning, neural networks, random forests, temperature prediction

## Abstract

This study describes accurate, computationally efficient models that can be implemented for practical use in predicting frost events for point-scale agricultural applications. Frost damage in agriculture is a costly burden to farmers and global food security alike. Timely prediction of frost events is important to reduce the cost of agricultural frost damage and traditional numerical weather forecasts are often inaccurate at the field-scale in complex terrain. In this paper, we developed machine learning (ML) algorithms for the prediction of such frost events near Alcalde, NM at the point-scale. ML algorithms investigated include deep neural network, convolution neural networks, and random forest models at lead-times of 6–48 h. Our results show promising accuracy (6-h prediction RMSE = 1.53–1.72°C) for use in frost and minimum temperature prediction applications. Seasonal differences in model predictions resulted in a slight negative bias during Spring and Summer months and a positive bias in Fall and Winter months. Additionally, we tested the model transferability by continuing training and testing using data from sensors at a nearby farm. We calculated the feature importance of the random forest models and were able to determine which parameters provided the models with the most useful information for predictions. We determined that soil temperature is a key parameter in longer term predictions (>24 h), while other temperature related parameters provide the majority of information for shorter term predictions. The model error compared favorable to previous ML based frost studies and outperformed the physically based High Resolution Rapid Refresh forecasting system making our ML-models attractive for deployment toward real-time monitoring of frost events and damage at commercial farming operations.

## 1. Introduction

Damage to agricultural crops from frost is a major economic issue for farmers around the world. Annual frost damage to crops in the United States accounts for more economic losses than any other natural weather hazard. For example, a single frost event in 1990 caused losses in excess of $700 million (Attaway, 1997; Snyder et al., 2005). Agricultural operations in New Mexico, where the diurnal temperature variability during the late spring can exceed 30°C, are particularly prone to such frost events. New Mexico supports a large stone-fruit and tree nut industry, accounting for $210 million in sales annually and 8.1% of the total agricultural sales in the state, second only to livestock and dairy products (USDA and NASS, 2019). Among naturally occurring plant species, it has been shown that increasing spring temperature fluctuations due to climate change could increase frost damage in the future (Augspurger, 2013). Despite greater risks, sales from New Mexico's fruit and nut industry nearly doubled from 2012 to 2017, underscoring the importance of these crops to the region (USDA and NASS, 2019).

Typical frost mitigation techniques include the use of wind machines, sprinkler systems, burning fuel, and the heating of enclosed spaces such as greenhouses. However, these systems are costly to implement and operate and can cut into the profit margins of the crop producer, which is particularly restrictive for the small-scale farmer. Further compounding this issue, the farmer implementing such a mitigation system must also consider correctly identifying a damaging frost event to avoid expenditures on operating these systems when they are not needed. Thus, accurate meteorological information and frost forecasts can be extremely valuable (Katz et al., 1982; Cerdá et al., 2018).

Traditional temperature forecasts are derived from numerical weather models. For example, the model used by the National Weather Service (NWS) enables provision of temperature forecasts at a 12-km spatial resolution (48-h MAE = 2.48–3.12°C) (Baars and Mass, 2005), but these are not suitable for use at the scale of a specific farm site. Forecasts are frequently downscaled to the location of interest, but may suffer in accuracy in complex or mountainous terrain (Eccel et al., 2007; Lundquist et al., 2008; Goger et al., 2016; Strachan and Daly, 2017). One such model in use by the National Weather Service (NWS) is the operational North American Mesoscale model (NAMeso), which provides temperature forecasts at a 12- and 4-km spatial resolution and was found to have significant warm biases (7 Kelvin) in regions of valley cold pooling and temperature inversion in Utah at 12-h lead times (Reeves et al., 2011). Other numerical models, such as the Weather Research and Forecasting (WRF) model, offer higher resolution forecasts, but are computationally costly and rely on large forcing datasets (Prabha and Hoogenboom, 2008) making them impractical solutions for individual farmers. Given that frost events are highly localized, often displaying varying rates of crop mortality within a single field, these larger scale numerical weather models may not be able to accurately represent frost patterns at the sub-field scale suitable for agricultural operations.

Machine Learning (ML) algorithms have been developed to detect frost events for frost forecasting as well as other meteorological applications (Kuligowski et al., 1998; Maqsood et al., 2003; Eccel et al., 2007; Lee et al., 2016). Use of ML methods trained on local data allows for the development of site-specific models to avoid factors such as the existence of complex terrain from impeding model accuracy as they do in existing larger scale numerical weather models. Previous studies where ML was employed to predict frost have yielded positive results in complex terrain (Ghielmi and Eccel, 2006; Eccel et al., 2007), and determined how integration of data from nearby weather station data may yield improved model predictions (Diedrichs et al., 2018). However, many ML frost prediction studies have either focused on classification of frost events (Möller-Acuña et al., 2017; Tamura et al., 2020; Noh et al., 2021) which, depending on the stage of bud development, may not be the most useful for characterizing actual crop mortality. For example, although the occurrence of frost may be enough to kill crops that are in the latter stages of bud development where flowers have started to form, temperatures lower than freezing are needed to destroy crops in earlier bud stages (e.g., bud swelling) (Salazar-Gutiérrez et al., 2016). As such, temperature and the severity of the frost, and not merely the identification of frost occurrence, is a better determinate of actual crop mortality (Warmund et al., 2008). Moreover, this means that temperature regression models are preferred over frost classification models for operational use in agriculture. Another missing aspect from previous studies is the use of prediction windows from both shorter (< 8 h) and longer (24–48 h) lead times to accommodate appropriate preparations for frost mitigation. Often, a single lead time is reported (Diedrichs et al., 2018) or model performance is significantly reduced at longer lead-times (Tamura et al., 2020).

In this study, we develop and evaluate temperature prediction models using both random forest (RF) and deep neural network (NN) models. We use meteorological data from a weather station in Alcalde, NM to predict the overnight minimum temperature at the weather station site at multiple prediction lead-times (6–48 h) and across seasons. Further, to test model transferability to new sites as well as the inherited learning of the models, we again train and test the models using temperature data from shorter data collection records at a nearby farm. We compare our results against forecasts made by the High-Resolution Rapid Refresh Model (HRRR). This study differs from those conducted previously concerning the use of ML models for frost detection by (1) application of ML regression models to predict temperature and not just the occurrence of frost, (2) comparing model performance at differing lead times and across seasons, and (3) providing an evaluation of the transferability of models to nearby sites. The models are designed to have a low computation cost, such that they can be run locally with limited computation cost relative to physically based models.

The purpose of this study includes the following:

- Design ML models to predict the minimum daily temperature at various lead-times and seasons for the purposes of frost prediction.
- Determine the effectiveness of both RF and DNN models for frost prediction compared to physics-based models.
- Evaluate the input parameter importance for RF models based on the calculated feature importance.
- Assess the effectiveness of transfer model learning through additional training and testing at a nearby farm site.

## 2. Materials and methods

### 2.1. Data collection

We used 10 years of historical data at an hourly temporal resolution from the Natural Resource Conservation Service weather station in Alcalde, New Mexico to train and test our ML models (Alcalde Annual Report 2021, 2021). The Alcalde site is located just East of the Rio Grande River, which provides the primary source of irrigation for agriculture in the region. The area is characterized as a high elevation desert environment, with the station situated in a valley at 1,700 m elevation and surrounding mountains reaching elevations of up to 4,000 m. The climate is cold semi-arid (Köppen Geiger Classification: BSk), with an annual precipitation of 232 mm/year. The high elevation and dry climate of the region leads to large daytime to nighttime temperature swings year-round. During the mid-to-late boreal Spring, this diurnal temperature variability reaches 30°C, making crops grown in the area prone to extensive frost damage.

The Alcalde weather station collects a variety of meteorological and soil measurements at an hourly frequency. The parameters we utilized for our frost prediction ML model included temperature, radiation, relative humidity, soil temperature, precipitation, wind speed, and wind direction. The full list of parameters used to develop features for ML models are provided in Table 1.

**Table 1:** The variables recorded by the Alcalde weather station and used as features in the machine learning (ML) modeling.

**Parameter**	**Abbreviation**	**Units**
Average temperature	TAVG.H	°C
Maximum temperature	TMAX.H	°C
Minimum temperature	TMIN.H	°C
Instantaneous temperature	TOBS.I	°C
Average wind direction	WDIRV.H	0–360°
Average wind speed	WSPDV.H	mph
Max. wind speed	WSPDX.H	mph
Average dew point	DPTP.H	°C
Average precipitation	PRCP.H	inches
Instantaneous relative humidity	RHUM.I	%
Average solar radiation	SRADV	Watts
Instantaneous soil temperature (~4 in.)	STO.I-1	°C
Average saturated vapor pressure	SVPV.H	kPa

Each variable is reported at hourly intervals. Abbreviations for ML analysis and units are also included.

Because the minimum hourly temperature is highly variable (SD = 10.8°C) and frost mitigation strategies in agriculture are often implemented on a daily basis, we created rolling 24-h windows over which we predict the minimum temperature for that window. The 24-h rolling window size effectively dampens the predicted temperature, providing accurate temperature predictions, robust features, and practical information for frost mitigation.

Additional temperature data was collected using both sensors at nearby Freshies of New Mexico, LLC (Freshies Farm) to test the transferability of the ML models developed using the Alcalde station data. Freshies Farm is located ~4 miles Northwest of the Alcalde station and resides within the same valley along the Rio Grande River. Approximately 2 years of temperature data collected at a frequency of 10 min was used for model transferability testing. Sensors placed at Freshies farm recorded temperature at 10 min intervals about 2 m above the ground. We use one of these sensors placed outside and away from obstructions (greenhouses, trees, etc.) to continue to train the models.

For performance comparison against physics-based models, we use temperature forecasts produced by the HRRR model. The HRRR is a physics-based ensemble analysis system for forecasting temperature and atmospheric systems at a 3 km spatial resolution with forecasts ranging from 0 to 24 h (Kalina et al., 2021; Dowell et al., 2022; James et al., 2022). Field-based validations of the HRRR 6-h forecast yielded a RMSE of about 2–3°C (James et al., 2022). Because of the similarities in spatial and temporal resolutions, the HRRR model offers a good comparison against our ML models as a benchmark for forecasts that are publicly available to farmers. We use the 1–6-h HRRR instantaneous temperature forecast data predicted at midnight for the period of 2015–2020 for the spatial grid cell containing the alcalde weather station and compare the performance against the ML models. We then calculate a minimum 6-h forecasted temperature using the 6 different predictions from 1:00 to 6:00 am. To account for elevational differences within the 3 km grid cell and the height of the observed temperature instrument (2 m), we correct the HRRR data for bias using the mean bias error between the Alcalde observations and the HRRR forecast. We show the bias-corrected performance of the HRRR forecast against the alcalde weather station observations.

### 2.2. Machine learning methods and analysis

ML model predictions were made using lead-times of 6, 12, 24, 30, 36, and 48 h to predict the minimum temperature. This means that a 12-h prediction for a 24-h period ending at 6:00 am would be based on a forecast generated at 6:00 pm of the preceding day. While there is some overlap in the prediction and observation windows for prediction periods < 24 h, these shorter-term prediction periods were selected because they can be critical to implementing accurate and cost-effective frost mitigation strategies for agriculture. We trained each of the models using the weather station data from Alcalde, NM. For each data parameter described in Table 1, we calculate the minimum, maximum, and mean of that parameter over the 24-h window and then input them as features into the ML models. Note that the observed values (TOBS, TMAX, TMIN, etc.) are reported as the maximum, minimum, and average temperature value over the observation frequency of the Alcalde weather station and then report the data at that frequency and “OBS,” “MAX,” and “MIN” here are the values recorded by the weather station. We generate statistics (max, mean, and min) of these values over rolling 24-h windows to further expand the data and allow the algorithms to be trained on the temporal aspects of the data. We trained the data on the oldest 85% of the dataset (*n* ≅ 3,100) and tested the data on the most recent 15% (*n* ≅ 550). The training for the Alcalde station data was performed using a time-series cross-validation using threefolds over the training dataset. The testing dataset was withheld from this training dataset altogether and assessed after training had been finalized.

We use the Root Mean Squared Error (RMSE), coefficient of determination (*r*^2^), and F1 score to assess model performance. The RMSE is a useful aggregate for the magnitude of model error while the *r*^2^ value represents the proportion of the variation in the observed values that is predicted by the modeled values. The F1 score is a measure of classification accuracy combining both the model precision (false negatives) and recall (false positives) and provides a measure of how the model will perform in a decision-making framework. For application in frost damage mitigation, we use 0°C as a frost threshold to calculate the F1 score. While we show this metric, the models themselves are regression models due to the variable temperature at which a buds may experience frost damage at different stages in development.

#### 2.2.1. Random forests

We developed a RF regression model using the sci-kit learn package in Python (Pedregosa et al., 2011). RF models use an ensemble decision tree classification system and can be used for either regression or classification. The RF method was developed by Breiman (2001), which has been expanded on and is described in depth by Biau and Scornet (2016). A random forest consists of a large number of decision trees that are constructed using indpendent random samples from the training dataset for each tree. The trees are then aggregated by a process known as bagging, wherein individual trees are generated using samples from the original dataset and then use averaging to decide on a final estimator.

The individual decision trees are constructed by splitting the input data parameters at various nodes parameters by maximizing the CART-split criterion. The CART-split criterion (Crawford, 1989; Biau and Scornet, 2016) takes the following form:


$$\begin{array}{l} L_{reg,n}\left(j,z\right)=\;\frac{1}{N_{n}\left(A\right)}\overset{n}{\underset{i=1}{\sum }}{\left(Y_{i}-{\bar{Y}}_{A}\right)}^{2}1_{X_{i}\epsilon A}\;\\ -\frac{1}{N_{n}\left(A\right)}\overset{n}{\underset{i=1}{\sum }}{\left(Y_{i}-{\bar{Y}}_{A_{L}\;}1_{X_{i}^{\left(j\right)}<z}-{\bar{Y}}_{A_{R}\;}1_{X_{i}^{\left(j\right)}\geq z}\right)}^{2}1_{X_{i}\epsilon A}\; \end{array}$$

where,

*L*_*reg*_, is the CART-criterion function to be maximized.

*n*, is the number of data samples.

*j*, is some value from 1 to the number of possible directions for splitting the data.

*z*, is the position of the cut along the *j*th coordinate.

*A*, is a generic cell or sample of the input data space. *A* create the pair.

*N*_*n*_(*A*) is the number of data points within *A*.

*X*_*i*_, is the input data for the *i*th tree sample.

*Y*_*i*_, is the target estimate of the *i*th decision tree.

${\bar{Y}}_{A}$, is the average of *Y*_*i*_ such that *X*_*i*_ belongs to *A*.

*A*_*L*_, is equal to {*X ϵ A*:*x*^(*j*)^<*z*}.

*A*_*R*_, is equal to {*X ϵ A*:*x*^(*j*)^≥ *z* }.

For each sample cell A, the optimal cut is performed by maximizing *L*_*reg*_ over the number of possible directions for splitting at each tree and node and the number of all possible cuts in A.

Here, we use a regression model to predict the 24-h rolling window minimum temperature. We used a sequence of steps to develop the RF model. First, we ran the model using the 24-h rolling window time series of the minimum, maximum, and mean of the raw parameters at the Alcalde station. Next, we extracted the Gini feature importance metric (Menze et al., 2009) of the model parameters at each prediction lead-time window to determine which parameters are relevant within the prediction model and eliminated the parameters of less importance, maintaining 90% of the calculated cumulative feature importance. Gini importance calculates each feature importance as the sum of the number of splits that utilize a feature as a ratio to the number of samples that are being split. The ability of RF models to rank the importance of features and parameters further allows us to determine which measurements are necessary when setting up a meteorological sensor network that could be used to develop and train ML models for temperature prediction. It also allows us to understand which aspects of the weather system might inform model performance and what processes might be relevant. We calculate and report the weighted feature importance of our input parameters in each of the RF models.

Next, we extracted features from the weather station data which allows us to perform dimensionality reduction on the time-series data by calculating statistical and spectral features from the previous *n* time steps and then feeding these features into the RF model (Mudunuru et al., 2018; Yuan et al., 2019). We extracted features using only the relevant parameters as determined by the previous model run. The expansion of the data at this step in the feature extraction is large enough such that elimination of some parameters is necessary before feature extraction to reduce the training time. Next, we ran the RF model using the extracted features and again extract each feature importance. This time, we eliminated only specific features (not parameters) with low importance based on the extracted feature importance, while retaining the 20 features of highest importance. We then ran the model an additional time using only the desired features to get our final model result. The feature extraction and dimensionality reduction allow us to optimize computing resources, reduce overfitting of the model, and obtain higher accuracy in temperature predictions.

#### 2.2.2. Neural network models

We developed two neural network model configurations; a fully connected deep neural network (DNN) and a convolution long-short term memory neural network (CNN). We developed both models using the Keras package in Python. NN models are constructed from a series of neural nodes, which contain a weighted value that adjusts based on the performance of the model and the signals or connections between nodes. Nodes are organized into layers with connections between nodes of each layer. The weights of each node and connection between nodes are optimized using a gradient descent optimization algorithm. The Nesterov-accelerated Adaptive Moment Estimation algorithm, or Nadam, is a gradient decent optimization algorithm that includes a Nesterov momentum element as an improvement over the classical momentum version of the algorithm (known as Adaptive Moment Estimation or Adam). Gradient descent optimization is a popular way to iteratively optimize an objective function, and in this case to optimize the neural networks parameters (node weights) to minimize the loss. Nadam is described by Dozat (2016). First, the gradient, *g*(*t*), for the previous step is defined using the previous time-step, *t –* 1.


$$\begin{array}{l} g\left(t\right)=f^{{}^{\prime }}\left(x\left(t-1\right)\right) \end{array}$$

Then the two moments, *m* and *n*, are defined in equation *X* using the gradient and hyperparameters μ and γ.


$$\begin{array}{l} m\left(t\right)=\mu \times m\left(t-1\right)+\;\left(1-\mu \right)\times g\left(t\right)\\ n\left(t\right)=\gamma \times n\left(t-1\right)+\;\left(1-\gamma \right)\times g{\left(t\right)}^{2} \end{array}$$

Then the moments are biased corrected to compensate for the fact that the moments are set to 0 at the start of the descent.


$$\begin{array}{l} \hat{m}=\left(\mu \times m(t)/(1-\mu \right)+\;\left((1-\mu \right)\times g(t)/(1-\mu ))\\ \;\hat{n}=\gamma \times n\left(t\right)/\left(1-\gamma \right) \end{array}$$

Finally, the parameter value is set for time *t* using the bias-corrected moments, $\hat{m}\;$and $\hat{n}$, as well as the hyperparameter α (learning rate parameter). ε is a small constant used to avoid negative parameter values.


$$\begin{array}{l} x\left(t\right)=x\left(t-1\right)-\left(\alpha \times \hat{m}\right)/\left(\sqrt{\hat{n}}+\varepsilon \right) \end{array}$$

Nadam is used to optimize the search of the parameter space until the optimal solution is found.

Adaptive Moment Estimation (or Adam) is similar to Nadam but lacks the Nesteroy momentum element. This difference manifests in the bias-corrected calculations of $\hat{m}$ and $\hat{n}\;$shown below:


$$\begin{array}{l} \hat{m}=m\left(t\right)/\left(1-\mu \right)\\ \hat{n}=n\left(t\right)/\left(1-\gamma \right) \end{array}$$

Our DNN model contains 13 dense layers of 39 nodes each, with an additional output layer of 1 node (20,320 total NN parameters). 39 nodes are used in each layer, a construction of one node per input parameter. 13 layers allows for a sufficiently deep NN that still minimizes overfitting through cross-validation. Multiple configurations of full connected layers and nodes were tested, but we found this configuration to produce the most successful DNN model. We used “elu” as our activation function, “nadam” as our optimizer, and “log-cosh” as our loss function.

The CNN model contains two one-dimensional convolutions layers (16 filters and 8 filters), one LSTM layer (8 nodes), and an additional two fully connected deep layers (10 nodes) with a dropout of 0.2 (3,963 total NN parameters). For the CNN model we use “relu” as the activation functions, “adam” as our optimizer, and “MSE” as our loss function.

The feature extraction and reduction process used in the RF modeling should be automated within the NN models. The construction of the NN nodes, layers, and weights implicitly develop optimized non-linear relationships between the input parameters and the desired output analogous to the features used in the RF models. However, the construction of the NN model does not inform the direct relationship between the input parameter and the output objective.

**Figure 1:**
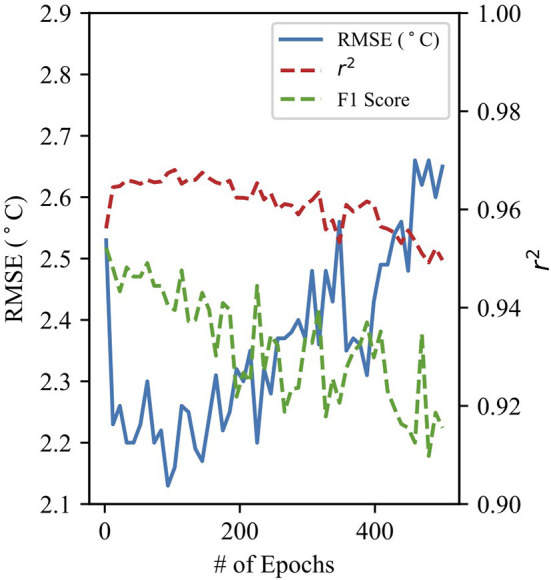
Performance statistics for the CNN 24-h prediction model as a function of the number of epochs used by the model.

The hyperparameters of both RF and NN models determine the structure and progression of the model and can be adjusted to optimize results. Figure 1 shows the CNN model performance against the number of epochs. The number of epochs determines how many times the entire dataset is passed through the model. The model RMSE and *r*^2^ improve as the number of epochs increases, but then deteriorate past about 100 epochs. This is likely due to the overfitting of the model to the training dataset at a large number of epochs. Hyperparameters for both RF and NN models were optimized to achieve a low RMSE when evaluating the model on the testing dataset and to minimize overfitting of the training dataset.

#### 2.2.3. Generalization

To test the generalization of the ML models, we continued training the models on the historical temperature data from a single sensor at Freshies Farm and tested those models using a separate subset of the Freshies data. By continuing to train the Alcalde models on the Freshies data, the models should be able to adapt to differences in the Freshies data while maintaining the patterns learned that are similar at each site, thereby requiring less training data. First, because only temperature data was available at Freshies Farm, we developed ML models from only the Alcalde hourly temperature parameters (TOBS, TMIN, TAVG, and TMAX; Table 1) constructed into 24 h rolling windows. Then, using the 10-min frequency temperature data collected at Freshies Farm, we resampled the data into hourly parameters used at Alcalde and constructed the same 24-h rolling windows. We then continued to train the models on the Freshies Farm data, initializing with the weights and forest structure of the ML models developed using the Alcalde data. Because of the limited amount of data collected at Freshies Farm, we perform only a single training-testing split when assessing the performance at Freshies Farm. Training the models on the Freshies Farm data allows us to determine the generalization of our ML models, while also providing practical information of the length of the temperature record needed to implement a sensor system integrated with accurate ML models for temperature predictions on-site.

## 3. Results

The results of the RF models compare favorably against previous temperature prediction efforts by machine learning models (Verdes et al., 2000; Diedrichs et al., 2018, 24-h F1 = 0.6–0.85, RMSE 2–3°C). Table 2 shows the test statistics at various prediction lead-times. The 6-h lead-time provided a temperature prediction with a RMSE of 1.72°C, while the 24-h prediction model resulted in a RMSE of 2.37°C. Predictably, the larger the prediction lead-time, the worse the model performed. The largest difference in RMSE between model runs occurs between 24 and 30 h (0.46–0.47°C), while the difference in RMSE between 36 and 48 h is relatively small (−0.02–0.17°C). The *rf* model does display some slight overfitting from training to testing set. However, the standard deviation in cross validation scores was small relative to the mean error, exhibiting a standard deviation of ±0.006–0.09°C RMSE and ±0.007–0.014 *r*^2^. The HRRR model 6-h forecast of minimum temperature resulted in a RMSE error of 3.05°C, a *r*^2^ value of 0.920, and a F1 score of 0.859 after correcting for bias.

**Table 2:** Statistics for the results of the RF model prediction at various lead-times.

**Random forest model statistics**						
**Prediction size (forecast)**	**Train RMSE [**°**C]**	**Test RMSE [**°**C]**	**Train** *r*^2^	**Test** *r*^2^	**Train** ***F*****1**	**Test** ***F*****1**
6 h	1.56	1.72	0.98	0.98	0.96	0.95
12 h	1.86	2.07	0.98	0.97	0.94	0.94
24 h	2.12	2.37	0.97	0.96	0.94	0.94
30 h	2.45	2.83	0.96	0.94	0.93	0.93
36 h	2.6	3.01	0.95	0.94	0.92	0.92
48 h	2.86	3.16	0.94	0.93	0.91	0.92

Statistics shown include root mean square error (RMSE °C), correlation coefficient (r^2^), and the F1 score. The training statistics for RMSE and r^2^ are the mean of 3 cross-validation folds.

The results of the fully connected DNN models are similar to that of the RF models and are reported in Table 3. The 6-h DNN prediction model resulted in a RMSE of 1.58°C and a F1 score of 0.94. The standard deviation across the threefolds of cross validation was also small relative to the mean error, ranging from ±0.014 to 0.029°C RMSE and ±0.004–0.01 *r*^2^. At shorter lead-times, the DNN model tended to have a lower RMSE than the RF model but performed worse at 30- and 36-h prediction intervals. The DNN models resulted in an equal or better *r*^2^ value compared to RF models, while the RF models resulted in an equal or better F1 score. Table 4 shows the test statistics for the CNN model. The CNN model again performs similarly to the RF and DNN models but exhibits slightly less overfitting than those models. The standard deviation in cross validation metrics was also comparable with ±0.007–0.09°C RMSE and ±0.003–0.01 *r*^2^. In the following figures, only the CNN results are represented because of the similarities between the CNN and DNN model results.

**Table 3:** Same as Table 2 but for the results of the fully connected DNN model predictions.

**Fully connected deep neural network model statistics**						
**Prediction size (forecast)**	**Train RMSE [**°**C]**	**Test RMSE [**°**C]**	**Train** *r*^2^	**Test** *r*^2^	**Train F1**	**Test F1**
6 h	1.55	1.53	0.99	0.98	0.96	0.96
12 h	1.72	1.84	0.98	0.98	0.96	0.95
24 h	2.06	2.23	0.97	0.96	0.95	0.94
30 h	2.34	2.69	0.96	0.95	0.94	0.92
36 h	2.53	2.96	0.96	0.95	0.94	0.91
48 h	2.7	2.94	0.95	0.94	0.94	0.93

The DNN model uses a sequential model with elu activation.

**Table 4:** Same as Table 2 but for the results of the CNN model predictions.

**Convolution LSTM neural network model statistics**						
**Prediction size (forecast)**	**Train RMSE [**°**C]**	**Test RMSE [**°**C]**	**Train** *r*^2^	**Test** *r*^2^	**Train F1**	**Test F1**
6 h	1.57	1.57	0.98	0.98	0.95	0.96
12 h	1.83	1.88	0.98	0.98	0.95	0.95
24 h	2.18	2.19	0.97	0.97	0.94	0.95
30 h	2.42	2.66	0.96	0.95	0.94	0.93
36 h	2.57	2.89	0.95	0.95	0.93	0.92
48 h	2.82	3.06	0.95	0.94	0.93	0.93

The CNN model uses two convolution layers and one LSTM layer configuration.

The *r*^2^ values show that the models are well correlated with the Alcalde weather station observations (0.92–0.98), and that the models are effective in capturing the variability of the minimum temperature. However, for the model to be operational, it must predict the temperature within an accuracy that can inform decision making. The models perform generally well at classifying the frost threshold (0°C, F1 = 0.9–0.95) and perform similarly well within the 6- to 24-h window of prediction (F1 = 0.94–0.95). The recall and precision scores were found to be comparable (test recall = 0.935–0.979, test precision = 0.846–0.949) with a slight preference toward false positive type error. This is encouraging given that the dataset is not perfectly balanced and contains only about 20% of values below the 0°C threshold. High recall scores suggest that the model is not simply predicting the majority class. This presence of slightly higher recall scores than precision scores is consistent across RF (recall = 0.96–0.98, precision = 0.88–0.94) and CNN (recall = 0.939–0.980, precision = 0.892–0.945) models.

**Figure 2:**
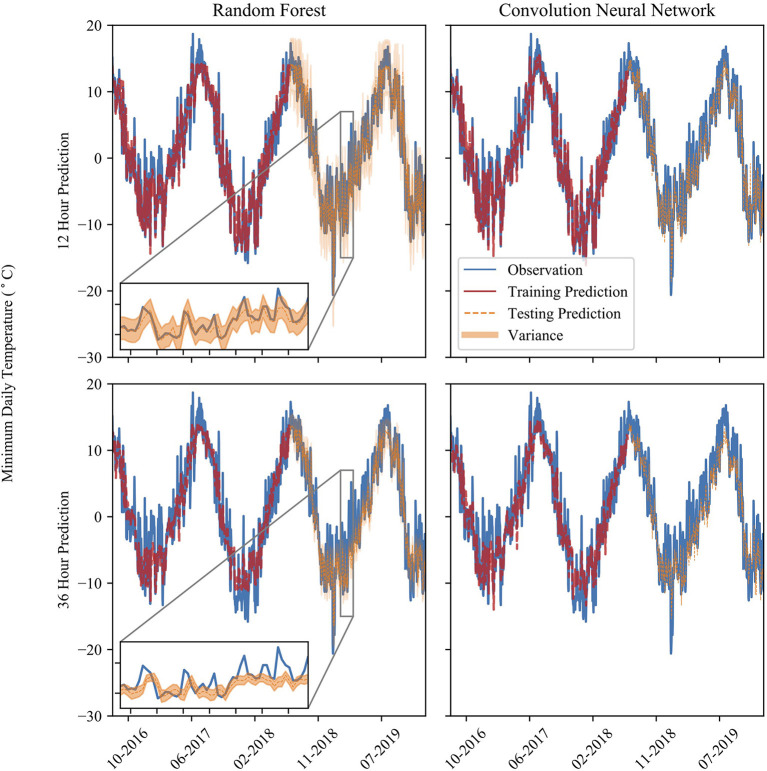
The time series of temperature prediction and observation at various prediction times (over a span of 3 years) and models. Model predictions show both training and testing datasets. The inset shows a period of 50 days from 01/29/2019 to 03/20/2019. The orange band is the 90% confidence interval of the RF model predictions.

The model experiences larger error when predicting for the local maxima of minimum temperatures, indicating they could experience lower classification ability at a warmer minimum temperature threshold. This is evident in Figure 2, which shows the chronological model predictions lead-times. Each of the models underestimates local maxima, while maximizing precision and sacrificing recall. This is especially evident at larger lead-times, where the model fails to capture the variability in the minimum temperature that shorter prediction lead-time models capture. Furthermore, the NN model shows a larger amount of variability in its predictions than the RF model. The RF model, especially at longer lead-times, captures the longer-term trends but fails to capture the day-to-day variability seen in the observations.

**Figure 3:**
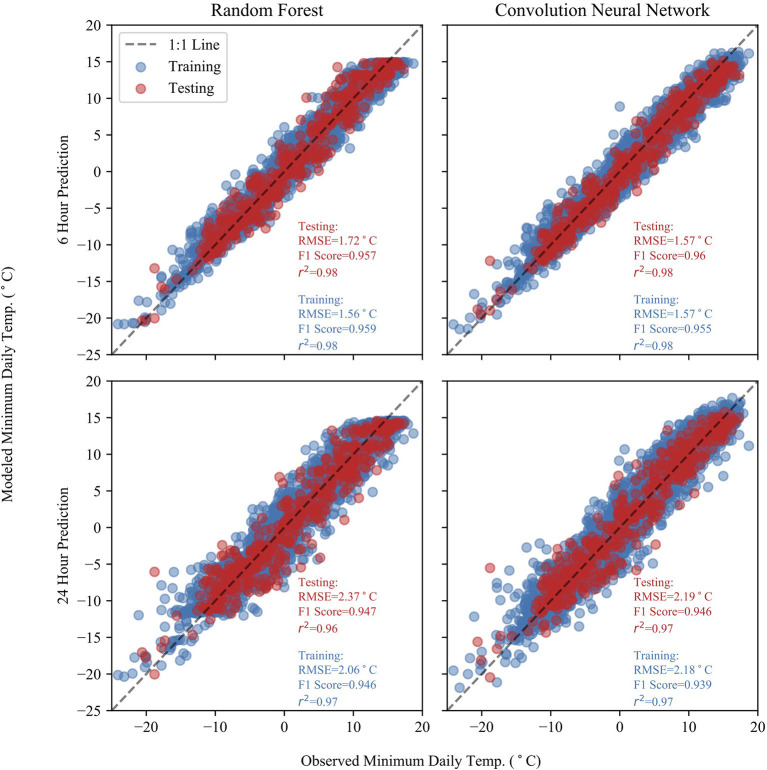
Observed and predicted temperatures at 6- and 24-h lead-times for both the RF and CNN models. Statistics for the predictions are included for each plot for both testing and training datasets. The predictions show good accuracy with low RMSE values.

Figure 3 shows the observed temperature against the model predicted temperature for both RF and CNN models at 6- and 24-h lead-times. The CNN model slightly outperforms the RF model, and the RF model fails to predict minimum temperatures above 15°C. Furthermore, the RF model exhibits biases at both high and low minimum temperatures, tending to overestimate at low minimum temperatures and slightly underestimate at the highest end of minimum temperatures. The RF model does capture the minimum temperatures well, but is outperformed by the NN model where there is sparser data at the extrema.

**Figure 4:**
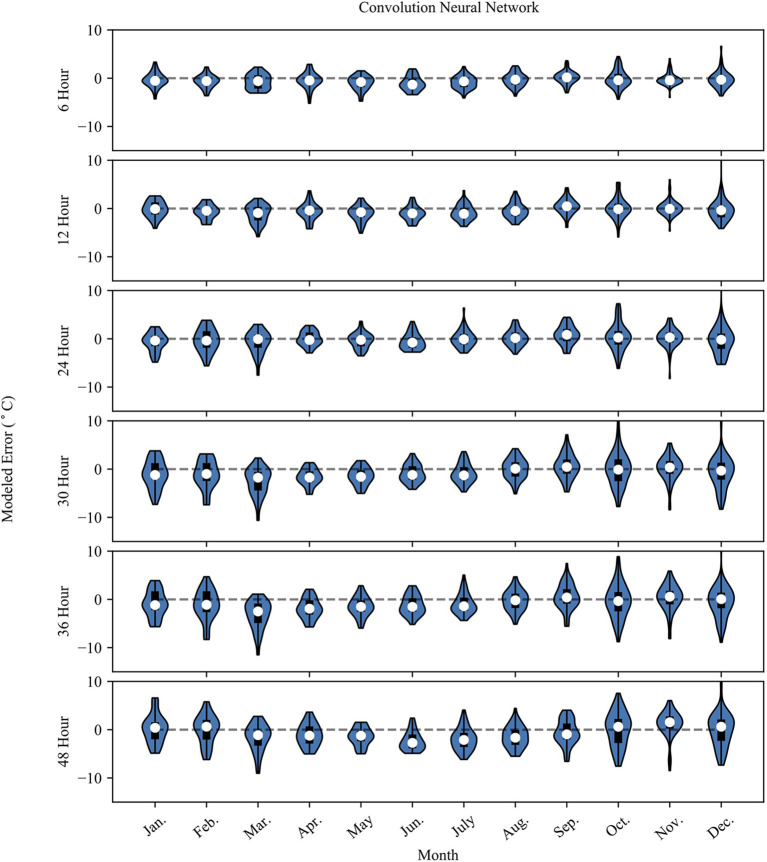
The plot shows the NN model error (Error = modeled – observed) grouped by month. The white dot represents the mean error for the month, the dark box shows the 25th and 75th percentile. The whiskers extend to the minimum and maximum possible error values. The surrounding blue polygon represents the probability density of the model error.

**Figure 5:**
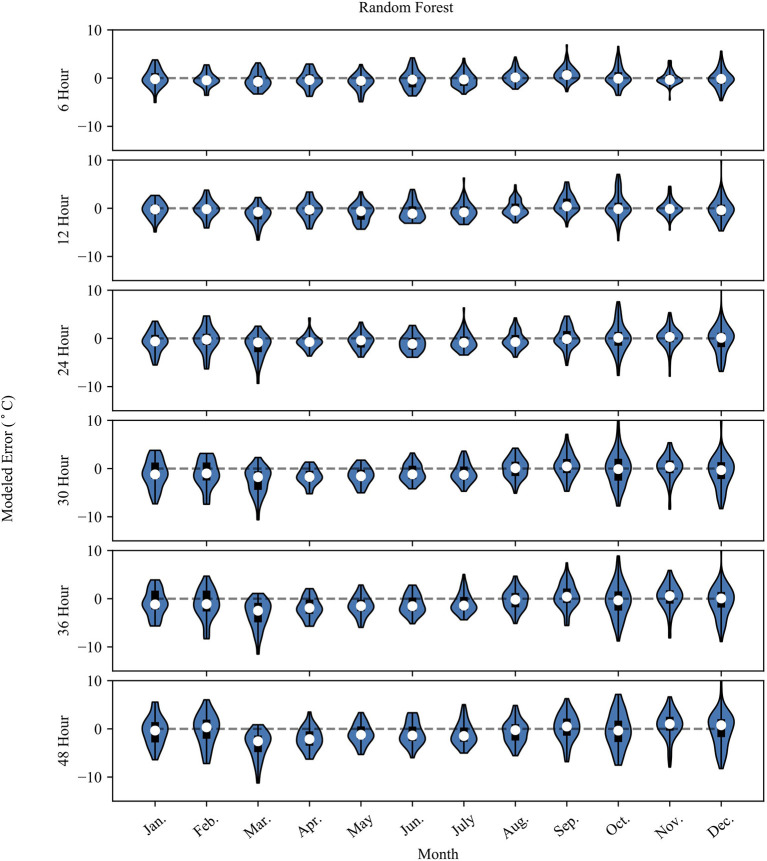
Same as Figure 4 but for the RF model results.

Figures 4, 5 show the monthly probability density distributions of model error for NN and RF models, respectively. The cooler months (Oct.–Mar.) for both models show longer tapered distributions at lead-times >12 h, while the warmer months (Apr.–Sep.) show more concentrated error distributions. The warmer months also feature a negative bias, especially for longer prediction lead-times.

**Figure 6:**
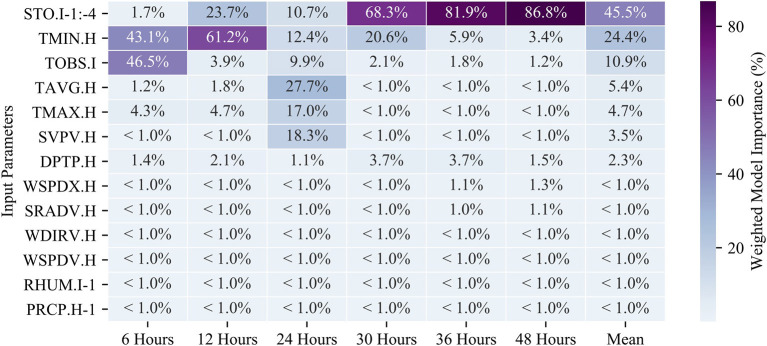
A heatmap of the weighted model importance of the RF model features (as described in Table 1). The listed values are the sum of all feature importances for the given input parameter (the actual number of features is much larger after feature engineering on the time-series data). Each RF model (columns) add up to a total of 100%.

A key functionality of RF modeling is that we can extract the feature importance (imp) used to determine branching within the model. Here, we report the Gini importance metric which is the number of times a feature is used to split a node weighted by the number of samples split by the node (Menze et al., 2009). Effectively, this indicates which input parameters are most useful to predicting the temperature at various lead-times in the RF models and gives us insight into which aspects of the weather system may be influencing the models. Figure 6 is a heatmap showing the weighted importance of each of the input parameters at different prediction intervals (refer to Table 1 for parameter abbreviations). The minimum hourly temperature (TMIN.H) and the observed instantaneous temperature (TOBS.I) provided the large majority of information used in the 6-h prediction model. The instantaneous temperature (TOBS.I, imp = 0.47) provides slightly more information than the minimum hourly temperature (TMIN.H, imp = 0.43). All other features that were tested provided minimal information toward predictions (imp < 0.05), suggesting that the short-term predictions rely heavily on utilizing the most recent temperature measurements. Interestingly, the minimum temperature (along with other air temperature measurements) was deemed important for models with shorter timescales of < 24 h (imp > 43%) but provides far less information to predictions made 36 h or more in advance (imp < 6%). The longer timescale models instead rely on the soil temperature for predictions lead-times of 30 h or more (imp > 68%).

Results of the temperature-only ML models for both the Alcalde station and with additional training at Freshies Farm are shown in Table 5. The temperature-only model on the Alcalde data performs worse than the full parameter ML model. However, the reduction in model performance is modest and the temperature-only models are less prone to overfitting, experiencing little to no difference in performance between testing and training. Using the temperature-only models, the modeled temperature is still highly correlated with the Alcalde observations (*r*^2^ = 0.92–0.98). The results of training and testing the data on the temperature data from Freshies farm shows that the models perform much worse when tested on the Freshies data at lead-times >12 h. Only the 6-h model (RMSE = 1.93°C) provides a RMSE of < 3°C (*r*^2^ = 0.86–87). At every other prediction lead-time (12-h +), the model produces poor results ate Freshies Farm (*r*^2^ = 0.26–60) when compared with the Alcalde tested models (*r*^2^ = 0.92–0.97).

**Table 5:** Testing set statistic for both RF and CNN models using only the temperature data from the Alcalde station along with the results from the same model with additional training on the temperature data from Freshies Farm.

**Prediction size (forecast)**	**Convolution neural network**				**Random forest**			
	**RMSE [** **°** **C]**		***r*** ^**2**^		**RMSE [** **°** **C]**		***r*** ^**2**^	
	**Alcalde**	**Freshies**	**Alcalde**	**Freshies**	**Alcalde**	**Freshies**	**Alcalde**	**Freshies**
6 h	1.72	1.97	0.98	0.87	1.79	2	0.98	0.86
12 h	2.17	3.27	0.97	0.6	2.23	3.29	0.96	0.58
24 h	2.38	3.45	0.96	0.53	2.41	3.59	0.96	0.49
30 h	2.92	3.7	0.94	0.43	2.96	3.63	0.94	0.43
36 h	3.11	3.86	0.93	0.33	3.16	4.09	0.93	0.2
48 h	3.26	3.94	0.92	0.29	3.24	3.88	0.92	0.26

**Figure 7:**
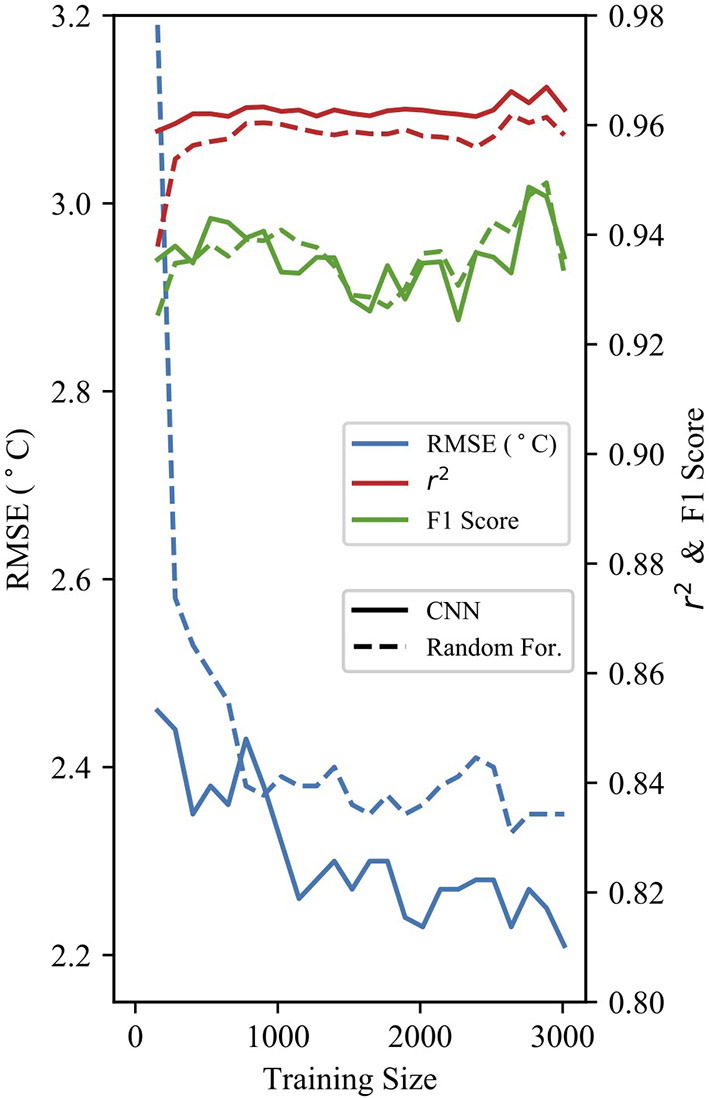
Performance statistics for both RF and CNN models at various training dataset sizes for 24-h predictions.

Figure 7 shows the model performance of both 24-h RF and NN full parameter models against the training size of the data. The models expectedly improve with greater the training data size. Dramatic reductions in RMSE were observed in both models with a training size of at least 1,000 samples. Above this training size, gains in model performance were minimal. The data record at Freshies Farm is < 3 years and thus not yet past the 1,000 sample threshold where we observe diminishing return from the Alcalde station data.

The computational time for each ML model type and prediction lead-time is reported in Table 6 (using a 16 GB RAM, i7-dual core, 2.8 GHz Laptop). The computational cost of the models is relatively low, especially in comparison with larger numerical weather models. The CNN models take by far the longest amount of time to train (205–264 s), while the DNN models take a slightly longer time to train (40–53 s) than the RF models (23–35 s). All three models take little time to run once they have been trained. For example, to run the DNN model on the entire Alcalde station time series (10 years of data) would take 0.03–0.11 s, while the CNN and RF models take 0.20–0.36 and 0.31–0.52 s to run, respectively. The low computational time demonstrated by these models would be reasonable to run on a smaller, field-based device such as a Raspberry Pi or Google TPU.

**Table 6:** The computational time of training and running the model on a single 1.7 GHz Intel Core i7 processor for each ML model type and prediction lead-time.

**Prediction size (forecast)**	**RF**		**CNN**		**DNN**	
	**Training time [s]**	**Run time [s]**	**Training time [s]**	**Run time [s]**	**Training time [s]**	**Run time [s]**
6 h	34.9	0.52	238.94	0.23	39.89	0.11
12 h	35.5	0.51	261.36	0.36	53.1	0.06
24 h	31.51	0.52	263.88	0.2	46.09	0.05
30 h	33.59	0.51	239.4	0.2	41.33	0.04
36 h	25.65	0.31	204.91	0.21	42.41	0.03
48 h	22.59	0.41	208.13	0.23	40.58	0.04

## 4. Discussion

The CNN and DNN model results slightly outperform the RF model results at each prediction lead-time. As the prediction lead-time increases, the RF model tends to dampen the temperature signal, showing less day-to-day fluctuation than the observed data. This is evident in Figure 5, where the probability density plot of the 48-g RF model prediction error is somewhat evenly distributed compared to the 6-h prediction where the distribution tapers away from the mean error in a more gaussian-like distribution. At longer timescales the RF model does an adequate job at capturing the longer-term trend, but fails to capture the day-to-day variability of the observed data as shown in the inset of the 36-h RF model in Figure 2. Meanwhile, the CNN model captures more of the variability of the observed data, displaying less overfitting, better model performance metrics, and less seasonal bias than the RF model. Thus, the CNN model is the preferred model in the context of this study. The results also show how the prediction accuracy may vary at different observed temperatures. Generally, the performance of both models is slightly worse above 10°C. The RF model predictions flatten at the highest observed temperature and does not predict temperature above about 15°C. This is also evident in the summer months, where the model underestimates summer temperatures.

Overall, the 6- and 12-h models provided promising results in frost mitigation applications at the Alcalde station (RMSE < 2°C, F1 > 0.95). Due to the localized nature of temperature in Northern New Mexico and the course spatial resolution of alternative temperature predictions (1 km-WRF, 3 km-HRRR, 12 km-NAMeso), the ML model predictions are likely an improvement over existing data available to farmers. The WRF and NAMeso models were found by one study to have a regional average RMSE of 2.6–2.88°C for a 24-h lead time (Raby et al., 2012), which is slightly worse than the model results for the Alcalde station (RMSE= 2.19–2.37°C). Further, The ML models presented outperformed the HRRR model forecast by a large margin, resulting in an improved 6-h forecast RMSE of about 1.5°C. Much of the improved performance is likely due to the scale of the prediction. ML models can predict the temperature at the point of the sensor, while large numerical based weather models generate forecasts at a much larger grid scale (3 km for HRRR). Numerical and physically based weather models are able to forecast weather patterns over a much larger area, which is useful for forecasting larger systems of weather (i.e., pressure fronts, storm events, etc.). However, for applications of frost damage where farmers require temperature data at the intra-field scale, point-scale forecasts from trained ML models may be more appropriate as demonstrated by this study.

Our models also compare well against previous ML studies with the purpose of frost prediction and mitigation (Diedrichs et al., 2018, 24-h F1 = 0.6–0.85, RMSE 2–3°C). However, the transfer learning results were not sufficiently accurate given the shorter data records at Freshies Farm (2 years). More data is needed at Freshies Farm (3+ years total) for further training of ML models before a frost mitigation system can be implemented. The relatively poor results of the transfer learning indicate that the models produced are location dependent and a large training data record is required to develop forecast models for specific locations. Theoretically, transfer learning can be used to transfer a pre-trained ML model to a new but similar location where data may be sparse. However, the two data collection sites in this study proved too dissimilar for such a method to achieve satisfactory results. Our results do indicate that given a sufficient sample size (1,000+ days) trained ML models can outperform traditional physically based model forecasts in an area of complex topography. Further, we expect the addition of key parameters, such as soil temperature, to improve model performance at longer lead times for Freshies Farm.

Use of RF models allowed us to determine the weighted importance of the various input parameters. The results show that many of the parameters reported by the Alcalde station are not used by the RF model to predict the temperature. Precipitation (PRCP), Relative Humidity (RHUM), Wind Direction (WDIRV), Wind Speed (WSPDV/WSPDX), and Solar Radiation (SRADV) each account for very little (~1% or less) weighted importance in the RF models at every prediction lead-time. Instead, the temperature parameters account for the vast majority of model importance, while atmospheric surface pressure provides some importance to the 24-h model (18.3%). As was shown for this instance, the variability in the barometric pressure is often associated with changes in temperature and has been frequently reported as a main drive of changes in local weather patterns (Rowson and Colucci, 1992). These parameter importances can also help to explain the relatively poor performance of the ML models at Freshies Farm at longer prediction lead-times. Comparing the model runs using the Alcalde station data and the Freshies Farm data, the best model performance at Freshies Farm is at the 6-h prediction lead-time when temperature data is the most relevant. At 24-h prediction lead-times, the Alcalde model relies on six separate input parameters, each exhibiting an importance of 9.9% or more. Beyond the 24-h lead-time, the Alcalde model relies most heavily on the soil temperature. Because these other parameters were not collected at Freshies Farm, ML models at longer lead times were still reliant exclusively on temperature and thus exhibited less robust model performance. Previous ML studies have also reported difficulty in predicting frost at longer intervals (Tamura et al., 2020) and the addition of soil temperature shows promise for model improvement over these prediction windows.

The differences in the models' monthly error distributions could be caused by the fundamental change to the weather patterns that occur during the Boreal Summer months. An important seasonal feature of the Southwest United States is the North American Monsoon (NAM), which is responsible for heavy summer rainfall across the region (Adams and Comrie, 1997). Higher atmospheric moisture during this time tends to dampen the diurnal signal of air temperature and could be responsible for seasonal differences in model accuracy. While the RF models attribute low importance to dewpoint (imp = 1.1–2.3%) and relative humidity (imp < 1.0%), those features could have a larger importance in seasonal models and have been shown to have higher model importance in other study locations (Ghielmi and Eccel, 2006; Eccel et al., 2007). Even still, the NAM activity is highest in July and August, while the largest negative model bias occurs during the Spring and early Summer months. Therefore, the cause of the strong negative bias could be associate with other factors outside of the NAM.

The highly localized variability in temperature indicated by our results is likely associated with the complex topography of the region. The topography in the vicinity of Freshies Farm is characterized by broad river valleys surrounded by steep escarpments forming mesas or table mountains. It is common for cool air to funnel from the mesas and pool in valleys in patterns related to the local topography, leading to highly localized spatial patterns in temperature (Burns and Chemel, 2014). Thus, while the Alcalde station and Freshies Farm are close geographically, the differences in temperature variability between sites may in part be due to different physical characteristics that control the microclimate of each site. Adding elevation or spatial data as well as other nearby meteorological data could contribute to better transfer of ML models to locations located within a larger network of farms and weather stations. The large datasets needed (3+ years) to train ML models makes implementing a frost prediction system at small farms where previous data may not exist difficult, but transfer learning may decrease the data collection period needed to implement such systems. Studies implementing transfer learning and data augmentation (Diedrichs et al., 2018) approaches have been minimal, and should be an emphasis for agricultural ML moving forward.

The models collectively take very little time to run (0.03–0.52 s) on a 16 GB RAM, i7-dual core, 2.8 GHz Laptop. The Raspberry Pi 4 specifications [8 GB RAM, Broadcom BCM2711, Quad core Cortex-A72 (ARM v8), 1.5 GHz] should be sufficient to run the ML models while being installed on local devices. Similar ML models in function and application run on a comparable laptop, took about 10 times the time to run on a Raspberry Pi 3 (35–40 s; 1 GB RAM, quad-core ARMv8, 1.2 GHz) compared to the laptop (3–5 s; 16 GB RAM, i7-dual core, 3.1 GHz) (Mudunuru, 2017). Thus, the computational cost and memory requirements of running pre-trained models (< 100 MB) should be small enough to be implemented in the field and run at the necessary frequency for frost event detection and prediction and present a large advantage over physically based weather models which require large computational power and would be impractical to run at the point scale for individual farmers. This approach could also apply to similar applications in edge computing where poor network quality or large observational datasets make local ML modeling and data compression extremely valuable (Hu et al., 2018).

In this study, we were able to develop new ML models for frost prediction at various prediction lead-times and tested the performance of each. Overall, our study shows that both CNN and RF models provide accurate forecasts that improved with the length of the data record. By producing temperature regression forecasts rather than frost classification forecasts, we were able to produce forecast data that is more relevant to crop damage from frost at various bud development stages. Previous studies that focus on forecasting the presence or absence of frost neglect the role that bud stage and hardiness play in eventual crop damage from frost (Warmund et al., 2008). The model results were comparable or better than efforts from previous ML frost forecast studies (Verdes et al., 2000; Diedrichs et al., 2018) (24-h F1 = 0.39–0.68), although a direct comparison between studies is difficult due to differences in methodology, input data, environmental settings of where the models were applied, and prediction lead-time. Our temperature predictions also achieved better performance metrics than available large-scale numerical weather models such as the HRRR model and compared to models assessed in previous studies (Raby et al., 2012). Such forecasts could provide accurate predictions of frost at the point scale in areas of complex topography where numerical models perform relatively poorly. Further, these models are computationally efficient such that they can be incorporated onto a sensor, IoT (internet of things) device, or local hub, providing greater flexibility regarding the configuration of the network connection or data stream from sensor to cloud.

In continuing this research, we plan to expand the methodology to a larger network of weather stations and incorporate topographic and spatial information into our predictions to better capture the effects of cold-air pooling over a broad spatial area and improve the results of transfer learning. Further, our models could be integrated within an IoT sensor network such that multiple sensors can be deployed within a single field to better capture frost variability at the sub-field scale and could be integrated into a local alarm system to alert Farmers of forecasted frost events.

## Data availability statement

The datasets presented in this article are not readily available because Data collected at individual farms is the property of the NMSBA grant holders and is not available for public release. Requests to access the datasets should be directed to CT, carltalsma@carbonsolutionsllc.com.

## Author contributions

CT, KS, and MM conceived and designed the study. CT carried out the analysis and wrote the initial draft. KS and MM provided edits to the original draft, provided critical insight, and gave methodological suggestions. KS secured the funding acquisitions and resources for this study. BC and MP collected, prepared, and curated the raw data. All authors discussed the results and approved of the final manuscript.
